# Growth factor-mediated augmentation of long bones: evaluation of a BMP-7 loaded thermoresponsive hydrogel in a murine femoral intramedullary injection model

**DOI:** 10.1186/s13018-019-1315-6

**Published:** 2019-09-05

**Authors:** Carl Neuerburg, Lena M. Mittlmeier, Alexander M. Keppler, Ines Westphal, Änne Glass, Maximilian M. Saller, Philipp K. E. Herlyn, Heiko Richter, Wolfgang Böcker, Matthias Schieker, Attila Aszodi, Dagmar-C. Fischer

**Affiliations:** 1 Department of General, Trauma and Reconstructive Surgery, University Hospital, Ludwig-Maximilians-University Munich, Marchioninistr. 15, 81377 Munich, Germany; 2 LivImplant GmbH, Starnberg, Germany; 3 Institute for Biostatistics and Informatics in Medicine and Ageing Research, Research Group Biostatistics, Rostock University Medical Center, Rostock, Germany; 4 Department of Traumatology, Hand- and Reconstructive Surgery, Rostock University Medical Center, Rostock, Germany; 5 LLS ROWIAK LaserLabSolutions GmbH, Hannover, Germany; 6 Department of Pediatrics, Rostock University Medical Center, Rostock, Germany; 7 Present Address: Department of Urology, University Hospital, Ludwig-Maximilians-University Munich, Munich, Germany

**Keywords:** Osteoporosis, Murine femoral intramedullary injection model, Bone morphogenetic protein 7 (BMP-7), BDI-hydrogel

## Abstract

**Background:**

Due to our aging population, an increase in proximal femur fractures can be expected, which is associated with impaired activities of daily living and a high risk of mortality. These patients are also at a high risk to suffer a secondary osteoporosis-related fracture on the contralateral hip. In this context, growth factors could open the field for regenerative approaches, as it is known that, i.e., the growth factor BMP-7 (bone morphogenetic protein 7) is a potent stimulator of osteogenesis. Local prophylactic augmentation of the proximal femur with a BMP-7 loaded thermoresponsive hydrogel during index surgery of an osteoporotic fracture could be suitable to reduce the risk of further osteoporosis-associated secondary fractures. The present study therefore aims to test the hypothesis if a BMP-7 augmented hydrogel is an applicable carrier for the augmentation of non-fractured proximal femurs. Furthermore, it needs to be shown that the minimally invasive injection of a hydrogel into the mouse femur is technically feasible.

**Methods:**

In this study, male C57BL/6 mice (*n* = 36) received a unilateral femoral intramedullary injection of either 100 μl saline, 100 μl 1,4 Butan-Diisocyanat (BDI)-hydrogel, or 100 μl hydrogel loaded with 1 μg of bone morphogenetic protein 7. Mice were sacrificed 4 and 12 weeks later. The femora were submitted to high-resolution X-ray tomography and subsequent histological examination.

**Results:**

Analysis of normalized CtBMD (Cortical bone mineral density) as obtained by X-ray micro-computed tomography analysis revealed significant differences depending on the duration of treatment (4 vs 12 weeks; *p* < 0.05). Furthermore, within different anatomically defined regions of interest, significant associations between normalized TbN (trabecular number) and BV/TV (percent bone volume) were noted. Histology indicated no signs of inflammation and no signs of necrosis and there were no cartilage damages, no new bone formations, or new cartilage tissues, while BMP-7 was readily detectable in all of the samples.

**Conclusions:**

In conclusion, the murine femoral intramedullary injection model appears to be feasible and worth to be used in subsequent studies that are directed to examine the therapeutic potential of BMP-7 loaded BDI-hydrogel. Although we were unable to detect any significant osseous effects arising from the mode or duration of treatment in the present trial, the effect of different concentrations and duration of treatment in an osteoporotic model appears of interest for further experiments to reach translation into clinic and open new strategies of growth factor-mediated augmentation.

**Electronic supplementary material:**

The online version of this article (10.1186/s13018-019-1315-6) contains supplementary material, which is available to authorized users.

## Background

In older adults, osteoporosis is the most common disease of the bone and will acquire increasing importance due to the demographic changes of our aging population [1]. Up to 27% of patients with a diagnosed osteoporosis already sustained a fracture [2]. Without any medicamentous therapy, patients with multiple osteoporosis-associated fractures will sustain in up to 85% another fracture [2]. The proximal femur fracture is one of the most common osteoporosis-associated fractures and often the consequence of an untreated osteoporosis [3]. It is known that for patients with an osteoporosis-associated femoral fracture the risk of sustaining a secondary fracture of the contralateral side is between 2.3 and 10.6% [4, 5]. Mortality of patients with a secondary femoral fracture during the first year after fracture is significantly increased compared to unilateral femoral fractures and ranges from 8.4 to 36% [5]. Sawahla et al. described that in two thirds of patients with secondary femoral fracture, the second fracture was at the corresponding anatomical location and that approximately 70% of second fractures occurred within 3 years [6].

Therefore, approaches for prophylaxis of secondary fractures should have the aim to counteract these developments. Current prevention measures aim at a strict compliance of the patients concerning medicamentous and non-medicamentous therapies, which is often difficult because of comorbidities such as cognitive disorders or dementia [7]. Local osteo-enhancement procedures as mentioned in the new “European guidance for the diagnosis and management of osteoporosis in postmenopausal women” could offer an alternative treatment for this problem [8]. These procedures comprise a minimal-invasive injection of a resorbable synthetic bone graft substitute, containing a proprietary triphasic calcium sulphate/calcium phosphate implant in the femoral neck, which leads to an increase of femoral BMD (bone mineral density) [8].

Strengthening the femoral neck with a thermosensitive, biocompatible material such as a hydrogel loaded with the growth factor BMP-7 could provide another opportunity.

Hydrogels are intensively investigated as carriers for biologicals in the context of tissue engineering [9, 10]. A wide used material on which many hydrogels are based on is Pluronic. Pluronic is a thermosensitive polymer, which is gelling when exceeding a critical solution temperature (LCST) [11, 12]. Furthermore, different chain extenders can modify Pluronic and its chemical properties. In this study, a Pluronic (P123)-based hydrogel (Europe Patent number: WO2014095915A1) was used, which was extended by butane diisocyanate (BDI) to have the desired properties, e.g., thermosensitivity. As shown in previous experiments by some of the authors, 1,4-butane diisocyanate extended triblock copolymer Pluronic P 123 (BDI-hydrogel) gels at body temperature and offers good biocompatibility and improved cell survival under in vitro and in vivo *circumstances* [12]. Its bioresorbable characteristics further provide an effective carrier for the subsequent release of growth factors.

BMPs are clinically used because of their potent osteoinductive stimulation [13]. This stimulation of bone formation becomes especially relevant under certain conditions, e.g., osteoporosis and fracture healing [14, 15]. BMP-7 gained approval for local treatment of distinct bone-related conditions, e.g., posterolateral spinal lumbar fusion and complicated permanent tibial pseudarthrosis [9, 10]. However, utilization of BMP-7 as a therapeutic drug poses a variety of problems. On the one hand, application has to be restricted to the desired site of action in order to avoid ectopic mineralization, and on the other hand biological half-life is in the range of a few minutes only [16]. Thus, either repeated applications are required or the BMP-7 has to be trapped inside a biodegradable matrix with subsequent slow and long-lasting release of the bioactive growth factor at the predefined site of action [17].

The present study therefore aims to test the hypothesis if a BMP-7 augmented hydrogel is an applicable carrier for the augmentation of non-fractured proximal femurs. To test this hypothesis, the murine femoral intramedullary injection model was used in the present pilot study (proof of principle) to gain knowledge on the biocompatibility and features of a BMP-7-loaded BDI-hydrogel in vivo*.*

## Methods

### Preparation of butane-diisocyanate hydrogel (BDI-hydrogel)

The synthesis of the BDI extended, thermoresponsive Pluronic 123 polymer has been previously described by Volkmer et al. [12]. For preparation of the hydrogel, 1 g of autoclaved polymer was dissolved under aseptic conditions in 9 ml of minimum essential medium alpha with l-glutamin (MEMalpha; Invitrogen, USA) supplemented with 10% fetal bovine serum (FBS; Sigma, Germany) and 40 IU/ml penicillin/streptomycin (PAA Laboratories GmbH, Austria), stored at 4 °C and gently stirred once per day [12]. At the day of surgery, rhBMP-7 (Olympus Biotech Corporation, Mt Waverley, Victoria, Australia) was added to yield a final concentration of 100 μg/ml rhBMP-7, and the solution was stored on ice until application.

### Animals and surgical procedure

Thirty-six male C57BL/6 mice were delivered at 6 months of age from Charles River Laboratories, Inc. (Sulzfeld, Germany), and all animals had access to maintenance diet and water ad libitum. Mice were kept in individually ventilated standard cages (6 mice per cage) in a temperature-controlled room on a 12-h light/dark cycle. After 2 weeks of acclimatization, mice were randomly allocated to three experimental groups, i.e., femoral intramedullary injection of either 100 μl saline (Sham group, *n* = 12), 100 μl BDI-hydrogel (Hydrogel group, *n* = 12), or 100 μl BMP-7 loaded BDI-hydrogel (BMP-7 group, *n* = 12) with scheduled observation periods of 4 and 12 weeks. Surgery was done at the right hindlimb in all animals. Anesthesia was achieved by intraperitoneal injection of fentanyl (0.05 mg/kg body weight (BW)), midazolam (5 mg/kg BW), and medetomidine (0.5 mg/kg BW). Surgery was performed as described by Zilber et al. except utilization of a dental drill to get access to the intramedullary cavity [18]. Thus, surgical approach was provided via a 5-mm anterolateral skin incision to expose the patellar tendon. Via blunt dissection of the tendon surrounding tissue and joint capsule, the distal aspect of the femoral shaft was then exposed. The intramedullary space was cannulated with a 29G needle and the compounds mentioned above were delivered to the proximal femur by means of a syringe. The incision was closed using a 4-0 Ethilon skin suture (Ethicon, Sommerville, NJ, USA), and anesthesia was antagonized with naloxon (1.2 mg/kg BW), flumazenil (0.5 mg/kg BW), and atipamezol (2.5 mg/kg BW) dissolved in 0.9% saline, each. Animals were placed under a red light until fully awake.

At the end of the scheduled observation period, animals were sacrificed in a CO_2_ chamber. Both hindlimbs were extracted; the femora were freed from muscle and adjacent tissue and immediately fixed in 10% formalin for 24 h. Thereafter, bones were incubated in 70% ethanol (24 h) and stored in 80% ethanol until further analysis.

### High-resolution three-dimensional X-ray tomography (μCT) of the femora

**Fig. 1:**
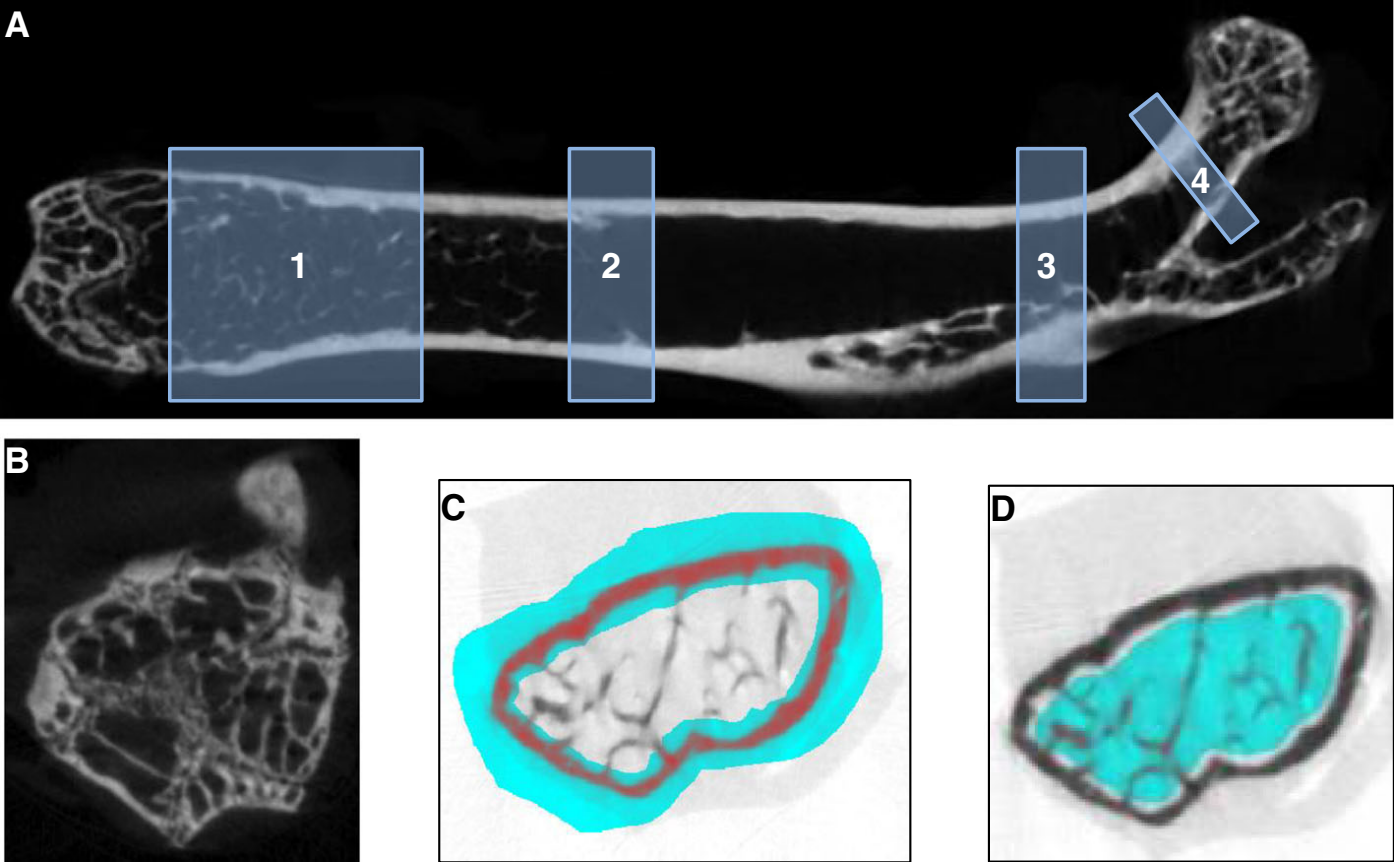
Schematic representation of the VOIs selected for analysis of cortical and trabecular bone microarchitecture (**a**), the distal growth plate as an example of an anatomical landmark (**b**), representative images of the manually drawn ROIs for analysis of cortical (**c**), and trabecular (**d**) bone. The distal growth plate and the division into greater trochanter and femoral neck served as internal references, i.e., positioning of the VOIs for analysis of the distal and proximal femora as well as the femoral neck. For examination of the distal femur (1), VOI started 50 slices apart from the growth plate (**b**) and consisted of 100 slices. The VOI in the region diaphysis (2) started 400 slices apart from the growth plate and consisted of 100 slices. In case of the proximal femur (3), trab and cort VOIs consisted of 80 slices each and started 50 slices distal from the division into greater trochanter and femoral neck. For analysis of the femoral neck (4), cort VOI and trab VOI included 20 slices each and an offset of 15 slices from the division into greater trochanter and femoral neck was used

A high-resolution μCT-imaging system (Skyscan 1076, Bruker, Kontich, Belgium) was utilized essentially as described [19]. Briefly, femora were rinsed with distilled water twice, equilibrated with 0.9% NaCl (4 °C overnight), and wrapped in cling film, and up to three bones were placed sequentially into cylindrical polypropylene tubes. The tubes were held by means of a Styrofoam bedding with the long axis of the bones orientated orthogonally to the X-ray beam. The instrumental settings chosen for scanning, reconstruction (NRecon, Bruker), and image analysis (CTAn, Bruker) are given in Table 1. Distinct volumes of interest (VOI) for analysis of cortical (cort) and trabecular (trab) bone were defined relative to anatomical landmarks (Fig. 1). In particular, cortical and trabecular bone were investigated at the diaphysis (cort), the distal femur (trab), the proximal metaphysis (cort, trab), and femoral neck (cort, trab), respectively. For analysis of cortical bone, a manually drawn bagel-like region of interest (ROI) was defined whereas manually drawn polygonal ROIs were used for analysis of trabecular bone. Size and shape of the polygonal ROIs were adjusted manually every 10–30 transverse slices and adjusted automatically by the software over the entire VOI (Fig. 1). All ROI interpolations were checked visually before starting the CTAnalyzer software. Parameters used for the characterization of cortical and trabecular bone are given in Table 2 [20]. For calculation of bone mineral density, calcium-hydroxyapatite (CaHA) phantom rods containing 0.25 and 0.75 g CaHA/cm^3^ were used for calibration of the instrument [19].

**Table 1:** Setting of the μCT for scanning, reconstruction, and analysis of bone microarchitecture

Settings for	
Recording of 3D images	
X-ray	48 kV/200 μA
Filter	0.5 mm Al
Pixel size	9 μm
Pixel matrix	4000 pixel x 2672 pixel
Rotation step	0.6°
Frame averaging	3
Reconstruction	
Defect pixel masking	20%
Beam hardening	30%
Misalingment compensation	calculated individually
Analysis	
Tresholding of gray values	
Cortical bone	100 - 255
Trabecular bone	85 - 255
Removal of white speckles	< 30 voxel

**Table 2:** Parameters used for the description of cortical and trabecular bone

Characteristics of cortical bone	
BAr/TAr, %	bone area / tissue area
CtTh, mm	cortical thickness
CtBMD, g/cm^3^	cortical bone mineral density
Characteristics of trabecular bone	
BV/TV, %	bone volume fraction
TbN, 1/mm	trabecular number
TbTh, mm	trabecular thickness
TbSp, mm	trabecluar separation
DA	degree of aniostropy
TbBMD, g/cm^3^	trabecular bone mineral density

### Preparation of bone samples for histological and immunohistochemical analysis

#### Methyl methacrylate embedding of mineralized bone

Per group, two pairs of randomly selected femora were embedded in methyl methacrylate (MMA, Technovit 9100 Heraeus-Kulzer, Wehrheim, Germany) and subsequently used for immunohistochemical evaluation. Embedding in MMA and preparation of the required solutions were according to the protocol of the manufacturer except that the procedure was performed manually [21]. Briefly, intact femora were dehydrated with increasing amounts of ethanol (70%, 80%, 90%, 96%, each for 24 h at 4 °C followed by two incubations in 100% ethanol (each 12 h at 4 °C) and defatting with xylene (2 × 12 h at 4 °C). Subsequently, the tissue was sequentially soaked with a 1:1 mixture of monomer and xylene (24 h at 4°), with monomer containing hardener 1 at a concentration of 3.4 mg/ml (24 h at 4 °C), with destabilized monomer containing 0.08 g/ml of polymethylmethacrylate (PMMA) and 4 mg/ml of hardener 1 (3–5 days at 4 °C). Finally, each sample was placed in a labeled Teflon-made polymerization mold and covered completely with polymerization solution (approx. 2 ml per sample), i.e., 10 volumes of destabilized monomer containing PMMA (0.16 g/ml) and hardener 1 (10 mg/ml) were mixed with 1 volume of destabilized monomer containing 0.08 ml/ml hardener 2 and 0.04 ml/ml regulator immediately before use. To ensure optimal conditions for polymerization, a mild vacuum was applied prior to closing the molds. Subsequently, the samples were stored at − 7 °C until polymerization was completed (usually 3–5 days). Blocks were trimmed and pre-cut in the area of interest with a diamond saw (Buehler Isomet 1000, Esslingen am Neckar, Germany). The surface of the blocks were polished and mounted on Leica Xtra adhesive slides with a special cyanacrylic glue (LLS ROWIAK LaserLabSolutions, Hannover, Germany). Ten-micrometer sections were prepared with a laser microtome (TissueSurgeon, LLS ROWIAK LaserLabSolutions, Hannover, Germany).

#### Immunohistochemistry

For immunohistochemical analysis of mineralized bone, sections were deplasted with methylmethacrylate (24 h) followed by overnight incubation with Shandon xylene substitute (Fisher Scientific, Schwerte, Germany). Subsequently, the slides were rinsed with distilled water, incubated in 2 M HCl (1 h, 37 °C), rinsed again with distilled water and 0.1 M Tris-HCl (pH 3.7) prior to trypsinization (0.5 mg/ml in 1% CaCl_2_; 30 min, 37 °C) for antigen demasking. Afterwards, the slides were rinsed with 0.1 M Tris-HCl and endogeneous peroxidase was blocked by incubation with 10% H_2_O_2_ (30 min, room temperature). For blocking of unspecific binding sites and visualization of bound antibody, the Vectastain Kit Elite Universal (Vector Laboratories Inc., Burlingame, CA, USA) was used according to the instructions of the manufacturer and essentially as described previously [22]. For detection of BMP-7, slides were incubated (4°, overnight) with the protein-A purified polyclonal rabbit-anti-BMP-7 antiserum (ab#56023, Abcam, Cambridge, UK), which was diluted with blocking serum to reach a concentration of 5 μg/ml. Paraffin-embedded samples from healthy human kidney served as controls.

#### Paraffin embedding and histochemical analysis of demineralized bone

Per group, two randomly selected pairs of femora were decalcified manually (Usedecalc®, Medite, Burgdorf Germany) at 4 °C for a total of 2 weeks with daily changes of the decalcification solution. Finally, the samples were washed with deionized water, dehydrated, cleared, and embedded in paraffin. Tissue samples (5-μm sagittal sections mounted on Superfrost® slides) were prepared for Masson-Goldner trichrome staining essentially as described previously [22, 23].

### Statistical analysis

All data were processed using IBM SPSS Advanced Statistics 22.0. Values are expressed as mean ± standard deviation (SD) for the indicated number of samples. For each animal, an individual left-to-right normalization of the measurements obtained from femora was performed for quantitative analysis of bone microarchitecture.

Parameters describing trabecular and cortical bone (Table 2) were analyzed and compared with repeated measure ANOVA for factors “treatment” (i.e., sham, BDI-hydrogel, and BMP-7) and “duration” (i.e., 4 or 12 weeks) with post hoc least significant difference (LSD) test, as well as for the within-subjects factor “localization” (i.e., diaphysis (cort), distal femur (trab), proximal femur, and femoral neck) with deviation-contrast, employing a general linear model for repeated measures (GLM-Rep), if normality was given. Here, estimated marginal means (EMMs) with their standard errors (SE_EMM_) and the respective 95% confidence intervals (CIs) are reported. Spearman Rho (R) test was used to analyze the correlation between normalized parameters describing trabecular and cortical bone, respectively.

In case of non-normality, nonparametric analysis of variance was performed employing the Kruskal-Wallis test for unpaired samples and the Friedman test for paired data, respectively. If appropriate, subgroups were tested pairwise using the Mann-Whitney *U* test (unpaired samples) or the Wilcoxon signed rank sum test (paired data), two-sided.

Normal distribution of measurements was checked using the Kolmogorov-Smirnov test. *P* < 0.05 (Bonferroni-adjusted for multiple testing) was considered to be statistically significant.

## Results

### Animals

The postoperative course was uneventful; wound healing was without any complications and no animal had to be taken out of the experiment before the end of the observation period.

### Analysis of bone microarchitecture

For each mouse, characteristics of cortical and trabecular microarchitecture were determined at the distal and proximal femur as well as the femoral neck of both hindlimbs. Normalized data on distinct features of bone microarchitecture relative to predefined anatomical landmarks together with data on treatment and duration are available. In order to investigate intrasubject interactions between these variables, the GLM-Rep model was applied. The analysis revealed that normalized TbN (trabecular number), TbTh (trabecular thickness), and the degree of anisotropy were virtually unrelated to the region investigated and were even not affected by the mode or duration of therapy (Additional file 1: Table S1). By contrast, normalized TbSp (trabecular separation) at the femoral neck was significantly lower compared to the distal and proximal femur, respectively (Table 3). While normalized trabecular BMD (bone mineral density) determined at the distal femur and femoral neck were almost comparable, it was significantly lower at the proximal femur (Table 3). However, neither normalized TbSp nor normalized trabecular BMD was affected by mode or duration of treatment.

**Table 3:** Normalized TbSp, normalized TbBMD, and normalized BAr/TAr relative to the investigated part of the femur. Estimated marginal means (EMM) with their 95% CIs from GLM-Rep model are reported. Superscripts denote significant differences between groups labeled identically (^*^p < 0.05; ^**, ##^*p* < 0.01)

	EMM	SE_EMM_	95% CI
Normalized TbSp			
Distal femur	1.049^*^	0.022	1.003 - 1.095
Proximal femur	1.050^**^	0.021	1.007 - 1.093
Femoral neck	0.977^*,**^	0.022	0.933 - 1.022
Normalized TbBMD			
Distal femur	0.960^**^	0.023	0.911 - 1.008
Proximal femur	0.882^**,##^	0.027	0.825 - 0.938
Femoral neck	1.056^##^	0.063	0.924 - 1.187
Normalized BAr/TAr			
Diaphysis	0.988^**^	0.018	0.951 - 1.025
Proximal femur	1.028^*^	0.017	0.992 - 1.064
Femoral neck	1.097^**,*^	0.031	1.032 - 1.161

**Fig. 2:**
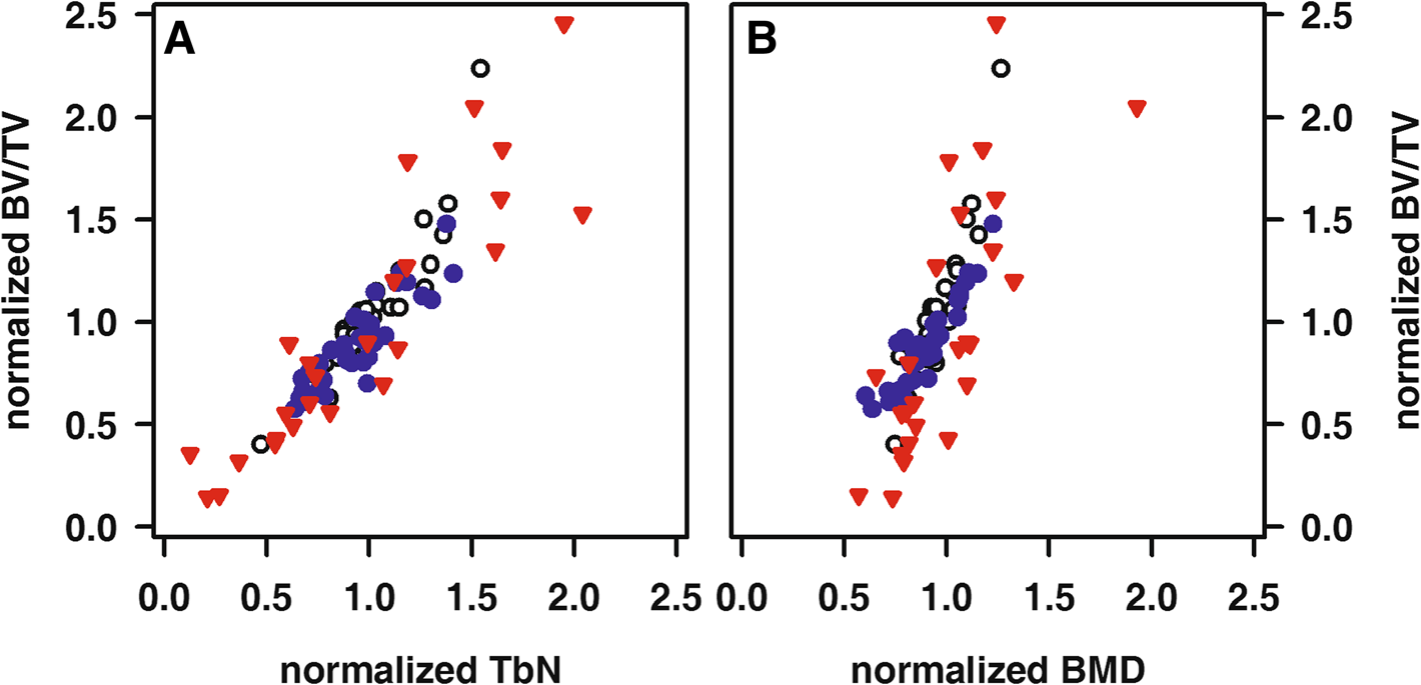
Significant association of normalized TbN (**a**) and normalized BMD (**b**) with normalized BV/TV determined at the femoral neck (red triangle), proximal (blue circle) and distal femur (white circle), respectively. Spearman’s rank coefficients of correlation: femoral neck, *R* = 0.94 (**a**) and *R* = 0.79 (**b**); proximal femur, *R* = 0.88 (**a**) and *R* = 0.90 (**b**); distal femur, *R* = 0.95 (**a**) and *R* = 0.88 (**b**); each *p* < 0.001

Within each of the anatomically defined regions, significant associations between normalized TbN and either normalized BV/TV (percent bone volume) or normalized BMD as well as between normalized BV/TV and normalized BMD were noted (Fig. 2). Furthermore, normalized BMD determined at the distal and proximal femur were significantly associated (*R* = 0.61, *p* < 0.001).

In case of cortical bone, the normalized BAr/TAr (bone area/tissue area) at the femoral neck was significantly higher compared to those determined either at the diaphysis or at the proximal femur (Table 3). Again, there were no effects related to the mode or duration of treatment. While the normalized CtBMD (Cortical bone mineral density) was unrelated to the region investigated or the mode of therapy, the linear model revealed a significant effect depending on the duration of treatment (4 vs 12 weeks, 1.001 (0.978, 1.023) vs. 0.988 (0.966, 1.010); *p* < 0.05). This effect is even more pronounced when the effects of localization and mode of treatment are not considered at all, i.e., the *U* test revealed *p* = 0.002. Correlation analysis revealed a positive association between the normalized CtBMD determined at either anatomical region (diaphysis and proximal femur, *R* = 0.75; diaphysis and femoral neck, *R* = 0.71; proximal femur and femoral neck, *R* = 0.64; each *p* < 0.001).

### Histological analysis and detection of BMP-7

**Fig. 3:**
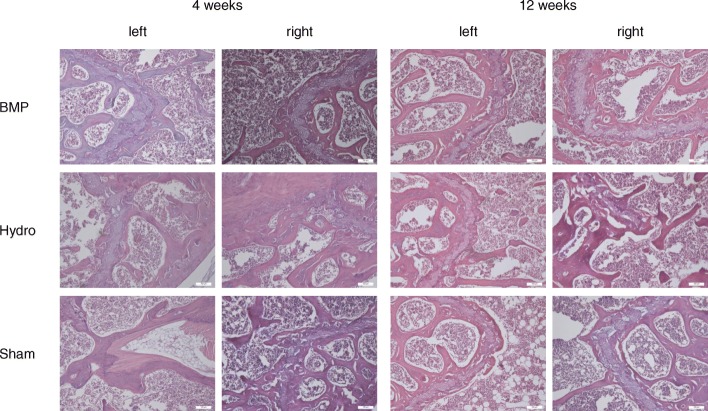
Hematoxylin-Eosin (HE)-stained sagittal sections of demineralized femora obtained 4 and 12 weeks after surgery. Femora on the right side were treated with either BMP-7 loaded BDI-hydrogel, intramedullary application of plain BDI hydrogel or saline (SHAM), the left sides serve as untreated controls. (Scale bar, 100 μm)

Histological analysis of demineralized paraffin-embedded bones did not reveal any signs of inflammation, such as clusters of granulocytes or inflammation foci. Neither, we could detect differences related to the mode or duration of the treatments (Fig. 3). No signs of necrosis could be detected throughout all specimens. Also, there were no cartilage damages, no new bone formations or new cartilage tissues.

**Fig. 4:**
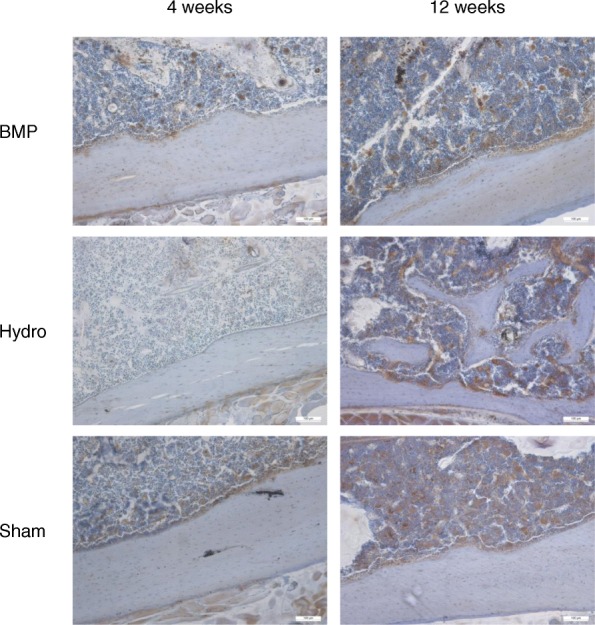
Immunohistochemical detection of BMP-7 in mineralized femora obtained 4 weeks (left column) and 12 weeks (right column) after intramedullary application of BMP-7 loaded BDI-hydrogel, plain BDI-hydrogel, or saline (SHAM) (Scale bar, 100 μm)

BMP-7 was readily detectable in all of the samples investigated (Fig. 4). In particular, there were no differences regarding the intensity of BMP-7-specific signals with respect to mode and duration of treatment.

## Discussion

Prophylactic augmentation of the proximal femur could offer an alternative strategy to reduce the high risk of secondary hip fractures associated with an underlying osteoporosis. This technique could be applied in non-fractured proximal femurs of patients undergoing surgery due to an osteoporosis-related fracture. While augmentation techniques available at present are based on either inert materials such as PMMA (Polymethylmethacrylate) or calcium-phosphate materials, the use of growth factor-augmented hydrogels could offer a new regenerative approach.

In order to evaluate the potential of a BMP-7-augmented hydrogel the present study was designed and conducted as a pilot (*proof of concept*) and with the vision for future utilization of a BMP-7-loaded hydrogel for pre-emptive augmentation of the femoral head in osteoporotic patients. A thorough investigation of this concept requires a sequence of well-designed animal experiments, covering all aspects from the feasibility of the surgical approach and biocompatibility up to toxicological and finally pharmacological effects. Our experiment was the first in this chain of events although no osteoporotic animals were used in the present in vivo analysis, we can clearly state that the mice tolerated the minimal-invasive surgical procedure well including assessment of the intramedullary cavity by means of a drill and concomitant application of the compounds. All mice were operated by the same surgeon and the postoperative recovery was without any complications. Neither μCT imaging nor histochemical investigation revealed any signs of surgically induced bone defects, inflammatory or reactive bone marrow changes secondary to the intervention.

Within the femur, we characterized bone microarchitecture by means of μCT imaging at four distinct anatomical regions, i.e. at the distal femur, the diaphysis, the proximal femur, and the femoral neck, and per mouse normalized data were generated. It is still a matter of discussion which part of the femur is best suited to characterize bone microarchitecture [24]. Furthermore, a general linear model for repeated measures was used to account for the factors “treatment” and “duration” as well as the within-subjects factor “localization”. This approach revealed that localization is relevant only for some of the parameters describing cancellous and cortical bone, respectively. In particular, BMD was lower at the proximal femur compared to the femoral neck or the distal femur, while TbSp was lowest and BAr/TAr was highest at the femoral neck. Both mineral density and trabecular microarchitecture, i.e., the spatial arrangement, contribute to stability. Results from the correlation analysis also point to a strong interaction between these two features. Furthermore, CtBMD decreased with aging. However, we could not detect increased bone formation secondary to local BMP-7 application.

In the present study, we applied for the first time (to the best of our knowledge) 100 μl pure or BMP-7-loaded BDI-hydrogel into the murine femoral cavity in vivo. Previous studies have shown that the BMP dosage is of importance with regards to osteoinduction. Yet, in literature, there is limited data on the concentration of BMP-7, whereas more information has been published on the concentration of BMP-2, which is why the choice of doses also considered reports on BMP-2 [25]. In the present study, we selected an amount of 100 μg/ml rhBMP-7. This concentration appeared reasonable in accordance to previous investigations in a small-animal study reported by Bosemark et al. in which a concentration of 25 μg BMP-7 was used [26]. Furthermore, in another dose-response study on BMP-7 mediated fracture healing utilizing a rat femoral critical size defect stabilized with a plate, the authors reported that a concentration of 50 μg was sufficient to induce bone healing [27].

A clear limitation of the experimental setup is the application of hydrogel through the distal femur. Although the hydrogel was applied as close to the femoral neck as possible, this does not guarantee that the hypothesized effects are restricted to the femoral neck. Further experiments by some of the authors on the properties of the hydrogel used compared to different hydrogels such as hexamethylene diisocyanate (HDI) and hydrogenated diphenylmethane diisocyanate (H12 MDI) showed that the BDI hydrogel as used in the present case provides the highest penetration resistance, which is of importance for injection of the biomaterial [12]. Also, previous studies of our group with cadaveric mice clearly showed a homogeneous distribution of the BDI-hydrogel within the femoral cavity (Additional file 2). 

Immunohistochemistry for detection of BMP-7 further showed that BMP-7 related signals were comparable between samples and most likely not related to the specific treatments. These findings are well in line with the data obtained by μCT imaging. Yet, in order to discriminate between exogenous and endogenous BMP-7, a tagged form, e.g., GFP-l or His-tagged rhBMP7 should be used in the future. Such an approach requires an additional set of controls and one has to ensure that the label itself does not interfere with the desired action of the growth factor and/or the designated analytical procedures.

Contrasting to our expectations, we were unable to detect any change in terms of bone morphology and mineralization, which suggests that further studies are necessary towards an optimization of the BMP-7 concentration. Previous unpublished in vitro analysis by our group showed a continuous release of rhBMP-7 from the gel over a period of 38 days. In light of these in vitro results, we expected a higher content of BMP-7 in the BMP-7-treated bones and an osteoinductive effect to be detected via μCT analysis.

Under physiological conditions, bone formation and degradation are well-balanced [28, 29]. Thus, one might not necessarily expect that the local application even of an extremely potent growth factor like BMP-7 will be able to overwrite the system of checks and balances in an otherwise healthy system. Therefore, this approach might reveal more robust results in a pathological rodent model, e.g., in a model of osteoporosis. Furthermore, we cannot rule out that we missed the peak, i.e., that there was an acute and intermediate effect within the first 1 or 2 weeks after surgery. Thus, for further analysis of this approach, a mouse model after induction of osteoporosis by for example orchiectomy or even with manifested disease appears to be more appropriate and represents the next logical step. Furthermore, by the time when the experiments were planned and performed, BMP-7 was provided on the market by Olympus Biotech Corp., which later in 2014, discontinued the sale of its products. Considering the recent market development with BMP-2 still on the market, an investigation of a BMP-2 augmented hydrogel would be desirable. The gel could also be loaded with other osteoinductive substances, e.g., anti-sclerostin antibodies.

## Conclusions

Augmentation techniques of the proximal femur could open the door for alternative strategies to reduce the high risk of secondary hip fractures associated with an underlying osteoporosis. Thus, growth factors such as BMP-7 could offer a promising opportunity to introduce osteoinductive properties in a resorbable hydrogel-based material. As evaluated in the present study, the murine retrograde injection model of the femoral bone appears to be feasible for further in vivo studies on the therapeutic potential on growth factor loaded BDI-hydrogel for preventing secondary osteoporotic femoral fractures in the future. Additional studies will be necessary to reach translation to clinical routine.

## Additional files

Additional file 1: **Table S1.** Normalized data on trabecular and cortical bone relative to the characteristics of therapy (treatment and duration) and localization of the VOI (mean±SD, *n*=6 each treatment group). (PDF 34 kb)

Additional file 2: Toluidine blue-labelled BDI-hydrogel injected into cadaveric femora shows homogenous dispersion of the material in the cavity. (TIFF 2150 kb)

## Data Availability

All data and materials were presented in the main paper and supplements attached.

## Abbreviations

ANOVA: Analysis of variance
BAr/Tar: Quantification of the trabecular bone fraction bone area/total area
BDI: Butan-Diisocyanat-hydrogel
BMD: Bone mineral density
BMP-7: Bone morphogenetic protein-7
BV/TV: Percent bone volume
BW: Body weight
CaHA: Calcium-hydroxyapatite
CtBMD: Cortical bone mineral density
EMMs: Estimated marginal means
GLM-Rep: General linear model for repeated measures
MMA: Methyl methacrylate
PMMA: Polymethylmethacrylate
ROI: Region of interest
SD: Standard deviation
TbN: Trabecular number
TbSp: Trabecular separation
TbTh: Trabecular thickness
VOI: Volumes of interest
μCT: X-ray micro-computed tomography
