# Supplementary material for: Growth factor-mediated augmentation of long bones: evaluation of a BMP-7 loaded thermoresponsive hydrogel in a murine femoral intramedullary injection model
Source: J Orthop Surg Res. 2019 Sep 5;14:297. doi: 10.1186/s13018-019-1315-6 (PMC6727400; doi:10.1186/s13018-019-1315-6)
Supplement: Supplementary file 1 — Table S1. Normalized data on trabecular and cortical bone relative to the characteristics of therapy (treatment and duration) and localization of the VOI (mean±SD, n=6 each treatment group). (PDF 34 kb) [file 13018_2019_1315_MOESM1_ESM.pdf]

		4 weeks			12 weeks			Overall
		Sham	BDI	BMP-7	Sham	BDI	BMP-7	
**trabecular bone**								
BV/TV	distal femur	0.98 ± 0.26	1.24 ± 0.57	1.12 ± 0.27	1.04 ± 0.18	0.92 ± 0.28	0.88 ± 0.14	1.03 ± 0.32
	proximal femur	1.01 ± 0.30	0.82 ± 0.14	0.94 ± 0.26	0.86 ± 0.06	0.90 ± 0.16	0.83 ± 0.22	0.89 ± 0.21
	femoral neck	0.85 ± 0.47	0.86 ± 0.67	1.29 ± 1.15	1.43 ± 0.98	1.00 ± 0.70	0.84 ± 0.37	1.03 ± 0.72
TbTh	distal femur	1.01 ± 0.07	1.08 ± 0.22	1.05 ± 0.10	1.02 ± 0.07	0.96 ± 0.09	0.99 ± 0.05	1.02 ± 0.11
	proximal femur	0.99 ± 0.08	0.99 ± 0.09	0.92 ± 0.14	0.96 ± 0.11	0.88 ± 0.07	0.94 ± 0.09	0.95 ± 0.10
	femoral neck	0.97 ± 0.41	0.83 ± 0.33	0.93 ± 0.24	1.74 ± 0.86	1.53 ± 1.19	0.85 ± 0.07	1.12 ± 0.70
TbN	distal femur	0.97 ± 0.22	1.10 ± 0.29	1.05 ± 0.21	1.01 ± 0.13	0.95 ± 0.27	0.88 ± 0.11	0.99 ± 0.22
	proximal femur	1.00 ± 0.23	0.82 ± 0.12	1.02 ± 0.22	0.90 ± 0.20	1.02 ± 0.17	0.89 ± 0.26	0.94 ± 0.21
	femoral neck	1.10 ± 0.66	0.79 ± 0.62	1.18 ± 0.86	1.18 ± 0.67	0.89 ± 0.55	1.01 ± 0.47	1.01 ± 0.60
TbSp	distal femur	0.98 ± 0.08	1.04 ± 0.15	1.03 ± 0.06	1.01 ± 0.11	1.08 ± 0.15	1.16 ± 0.07	1.05 ± 0.12
	proximal femur	1.03 ± 0.12	1.07 ± 0.12	0.98 ± 0.09	1.09 ± 0.12	1.00 ± 0.10	1.06 ± 0.15	1.04 ± 0.12
	femoral neck	0.91 ± 0.09	1.08 ± 0.13	0.91 ± 0.18	0.94 ± 0.13	1.00 ± 0.09	1.01 ± 0.09	0.97 ± 0.13

		4 weeks			12 weeks			Overall
		**Sham**	**BDI**	**BMP-7**	**Sham**	**BDI**	**BMP-7**	
TbDA	distal femur	0.85 ± 0.10	0.99 ± 0.39	0.89 ± 0.19	0.87 ± 0.19	0.82 ± 0.25	1.02 ± 0.07	0.91 ± 0.22
	proximal femur	1.06 ± 0.18	1.11 ± 0.30	0.93 ± 0.22	1.03 ± 0.14	0.97 ± 0.14	1.33 ± 0.28	1.07 ± 0.25
	femoral neck	0.54 ± 0.53	1.23 ± 1.47	4.49 ± 6.34	0.28 ± 0.11	0.88 ± 0.59	1.03 ± 0.96	1.38 ± 2.70
TbBMD	distal femur	0.98 ± 0.11	1.01 ± 0.16	0.98 ± 0.13	0.97 ± 0.07	0.94 ± 0.10	0.87 ± 0.06	0.96 ± 0.11
	proximal femur	0.98 ± 0.16	0.90 ± 0.09	0.91 ± 0.19	0.82 ± 0.10	0.90 ± 0.15	0.86 ± 0.15	0.90 ± 0.14
	femoral neck	1.10 ± 0.37	0.91 ± 0.16	1.08 ± 0.31	1.20 ± 0.52	1.07 ± 0.29	0.98 ± 0.20	1.05 ± 0.31

		4 weeks			12 weeks			Overall
		**Sham**	**BDI**	**BMP-7**	**Sham**	**BDI**	**BMP-7**	
**cortical bone**								
BAr/TAr	diaphysis	1.02 ± 0.11	1.02 ± 0.10	0.94 ± 0.08	0.91 ± 0.14	0.98 ± 0.11	1.01 ± 0.08	0.98 ± 0.10
	proximal femur	1.06 ± 0.09	1.07 ± 0.05	0.98 ± 0.09	1.02 ± 0.07	1.13 ± 0.12	0.94 ± 0.10	1.03 ± 0.11
	femoral neck	1.06 ± 0.28	1.14 ± 0.10	1.07 ± 0.16	1.19 ± 0.23	1.14 ± 0.18	1.07 ± 0.15	1.11 ± 0.18
CtTh	diaphysis	1.01 ± 0.08	0.99 ± 0.04	0.99 ± 0.04	0.96 ± 0.06	1.00 ± 0.07	1.02 ± 0.05	1.00 ± 0.06
	proximal femur	1.02 ± 0.01	1.00 ± 0.06	0.98 ± 0.04	1.02 ± 0.05	1.01 ± 0.05	0.99 ± 0.05	1.00 ± 0.05
	femoral neck	1.04 ± 0.11	0.99 ± 0.07	0.98 ± 0.04	1.01 ± 0.04	0.88 ± 0.23	0.99 ± 0.03	0.98 ± 0.12
CtBMD	diaphysis	1.02 ± 0.04	0.98 ± 0.04	0.99 ± 0.06	0.98 ± 0.02	1.01 ± 0.05	0.96 ± 0.02	0.99 ± 0.04
	proximal femur	1.03 ± 0.02	1.00 ± 0.03	0.99 ± 0.03	0.99 ± 0.03	1.02 ± 0.04	0.97 ± 0.02	1.00 ± 0.03
	femoral neck	1.05 ± 0.04	1.15 ± 0.33	0.98 ± 0.07	0.96 ± 0.03	1.00 ± 0.04	0.96 ± 0.04	1.02 ± 0.14
