# Supplementary figures and images for: Growth factor-mediated augmentation of long bones: evaluation of a BMP-7 loaded thermoresponsive hydrogel in a murine femoral intramedullary injection model
Source: J Orthop Surg Res. 2019 Sep 5;14:297. doi: 10.1186/s13018-019-1315-6 (PMC6727400; doi:10.1186/s13018-019-1315-6)

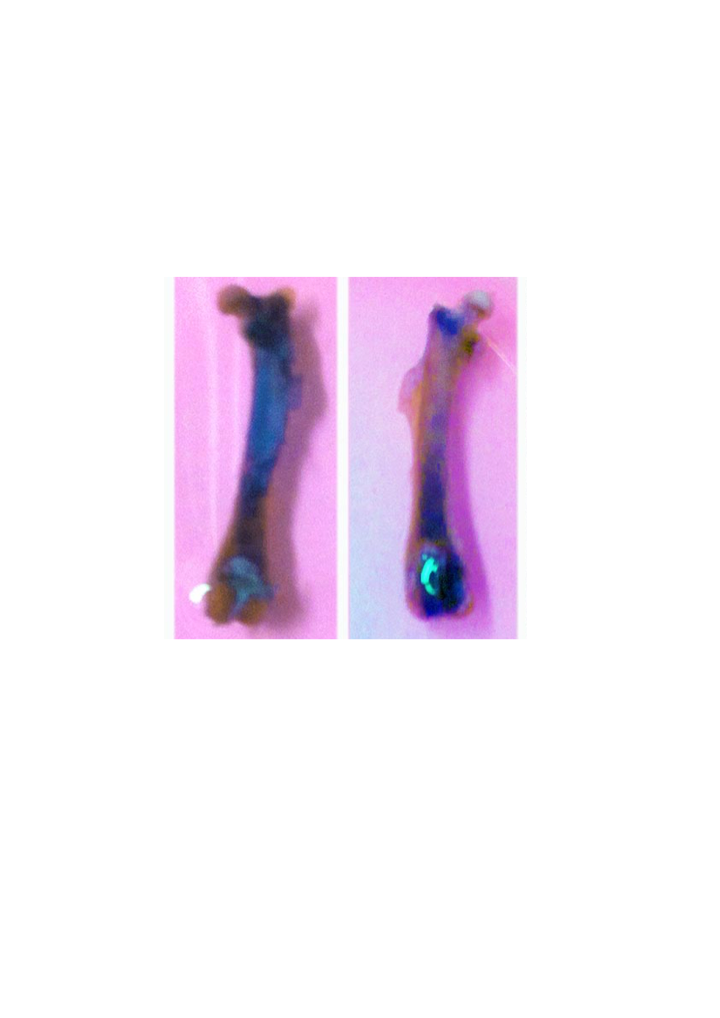

Supplement: Supplementary file 2 — Toluidine blue-labelled BDI-hydrogel injected into cadaveric femora shows homogenous dispersion of the material in the cavity. (TIFF 2150 kb) [file 13018_2019_1315_MOESM2_ESM.tiff]
